# mtDNA haplogroup and single nucleotide polymorphisms structure human microbiome communities

**DOI:** 10.1186/1471-2164-15-257

**Published:** 2014-04-03

**Authors:** Jun Ma, Cristian Coarfa, Xiang Qin, Penelope E Bonnen, Aleksandar Milosavljevic, James Versalovic, Kjersti Aagaard

**Affiliations:** 1 Departments of Obstetrics & Gynecology, Baylor College of Medicine and Texas Children’s Hospital, Houston, TX, USA; 2 Molecular and Human Genetics, Baylor College of Medicine and Texas Children’s Hospital, Houston, TX, USA; 3 Pathology and Immunology, Baylor College of Medicine and Texas Children’s Hospital, 77030 Houston, TX, USA

**Keywords:** HMP, Mitochondrial DNA haplogroup, Association, Microbiome, mtDNA SNP

## Abstract

**Background:**

Although our microbial community and genomes (the human microbiome) outnumber our genome by several orders of magnitude, to what extent the human host genetic complement informs the microbiota composition is not clear. The Human Microbiome Project (HMP) Consortium established a unique population-scale framework with which to characterize the relationship of microbial community structure with their human hosts. A wide variety of taxa and metabolic pathways have been shown to be differentially distributed by virtue of race/ethnicity in the HMP. Given that mtDNA haplogroups are the maternally derived ancestral genomic markers and mitochondria’s role as the generator for cellular ATP, characterizing the relationship between human mtDNA genomic variants and microbiome profiles becomes of potential marked biologic and clinical interest.

**Results:**

We leveraged sequencing data from the HMP to investigate the association between microbiome community structures with its own host mtDNA variants. 15 haplogroups and 631 mtDNA nucleotide polymorphisms (mean sequencing depth of 280X on the mitochondria genome) from 89 individuals participating in the HMP were accurately identified. 16S rRNA (V3-V5 region) sequencing generated microbiome taxonomy profiles and whole genome shotgun sequencing generated metabolic profiles from various body sites were treated as traits to conduct association analysis between haplogroups and host clinical metadata through linear regression. The mtSNPs of individuals with European haplogroups were associated with microbiome profiles using PLINK quantitative trait associations with permutation and adjusted for multiple comparisons. We observe that among 139 stool and 59 vaginal posterior fornix samples, several haplogroups show significant association with specific microbiota (q-value < 0.05) as well as their aggregate community structure (Chi-square with Monte Carlo, p < 0.005), which confirmed and expanded previous research on the association of race and ethnicity with microbiome profile. Our results further indicate that mtDNA variations may render different microbiome profiles, possibly through an inflammatory response to different levels of reactive oxygen species activity.

**Conclusions:**

These data provide initial evidence for the association between host ancestral genome with the structure of its microbiome.

## Background

Humans are remarkable hosts to microbes, and we have in fact co-evolved as highly plethoric communities. The NIH Roadmap initiative, known as the Human Microbiome Project (HMP) [1], enabled sequence-based comprehensive characterization of the adult human microbiota. Human-associated microorganisms are present in numbers exceeding the quantities of human cells by at least 10-fold beginning in the neonatal period, and the collective microbiome metagenome exceeds our human genome in terms of gene content by orders of magnitude (>150 fold) [2-4]. We now appreciate that the microbiota are a metabolically and antigenically vibrant diverse community, which may function as *mutualists* (symbiotically beneficial), *commensalists* (of neither harm nor benefit), or *pathogens* (detrimental to the host). However, we do not yet understand how the host genomic content influences its establishment.

The HMP Consortium [2-5] established just such a population-scale framework with which to characterize the relationship of microbial communities with their human hosts. The signature frameworks of as many as 18 body sites of 242 screened and phenotyped adults from the target population of 300 subjects have been described. To minimize exogenous and environmental exposure that may influence taxonomy abundance, rigorous clinical standards were applied to screen subjects to assure that the cohort were similar in baseline health status [4]. The majority of the subjects were non-vegetarian, non-smokers, non-obese and yet diverged with respect to race/ethnicity, and parental country of origin. Of interest, although no taxa were universally present among all body habitats and individuals, the carriage of metabolic pathways was surprisingly alike, with a greater degree of similarity observed among related race or ethnic groups [2,3]. These carriage patterns were functionally relevant, and genomic variation in microbial strains (gains, losses, and polymorphisms) underscored inter-individual variation in the microbiome. Taxonomic profiling associating both clades and metabolism with host covariates (namely age, gender, BMI, blood pressure, race and ethnicity, etc) demonstrated that most microbial variations are not well explained by examined clinical covariates other than race/ethnicity [2].

Race and ethnicity exert their effects through innate or genetically determined biologic mechanisms, and have broader implications with relation to socioeconomic status, diet habit, life style, etc. Therefore, it is not surprising to see the strong association of race/ethnicity with the microbiome. However, self-defined race/ethnicity is not always accurate and further complicated by secondary associations of race and ethnicity with diet, birth country, etc. Thus, further investigation down to the molecular level is essential to gain more knowledge on the underlying mechanism of association and to prevent potential misclassification bias.

One recent study has reported on both the commonality and the distinctions in the gut metagenome when compared among children and adults from rural Venezuela, Malawi, and the urban U.S. Of note, the study cohort was comprised of 531 subjects from a limited number of families (151). While relatively few distinctions in the gut microbiome were observed across all cohorts through the first 3 years of life, pronounced differences in the gut microbiome and functional gene repertoires were noted among geographic locations. However, there was no clustering observed among adult Malawians, Amerindians, nor regional U.S. populations. The investigators concluded that the host age/stage of development and geography served as primary determinants of the gut microbiome [6]. However, it bears mention that the potential for shared or divergent host ancestral genomic variation (mitochondrial or nuclear DNA) among their study cohorts was not investigated, and thus, this study may have failed to recognize an independent contribution of the host genome by examining likely homogenous populations (*e.g.,* 34 Malawian and 19 Venezuelan families with a significant number of mono and dizygotic twin pairs). Moreover, when analyzed by the degree of familial relatedness (mono and dizygotic pairs, siblings, and unrelated children and adults) and cohabitation, significant UniFrac distance metrics was observed with greater heterogeneity. Similar studies have not been duplicated in larger genetically divergent population-based cohorts living in the same region with relatively common diets and exogenous exposures [7].

In contrast, there are several lines of evidence suggesting that the host genomic ancestry may structure the microbiome. A large murine advanced intercross line detected a core measurable microbiota (CMM) consisting of 64 conserved taxonomic groups. In this cohort, 13 murine genomic regions and five quantitative trait loci significantly associated with 26 CMM taxa at both the genus and species level of operational taxonomic unit (OTU) projections [8]. With respect to humans, previous studies have largely utilized twin and unrelated sibling pairs as surrogates for host genomic identity [7,9,10]. Turnbaugh et al [7] employed 16S rRNA based analysis and observed that the gut microbial community structures of adult monozygotic (haplotype identical) twin pairs had a degree of similarity comparable to dizygotic pairs, and only slightly more similar to their mothers. Of note, there was a roughly comparable degree of covariation between adult monozygotic and dizygotic twin pairs and deviations from this “core” gut microbiome were associated with adult obesity [7,9,10]. Total community DNA and RNA was relatively deep sequenced from one set of obese monozygotic twins, and comparative analysis indicated that the majority of species-level projections were shared with relatively significant variations in abundance [10]. These findings expanded upon earlier unrelated infant and dizygotic twin pair studies across the first year of life, which similarly demonstrated that a higher degree of host relatedness was associated with akin gut microbiome profiles [11]. However, these studies were limited to associations with gut microbiota, failed to consider whether the host nuclear or mitochondrial genome (which is solely maternally inherited) might be driving these associations, nor controlled for the potential impact of perinatal co-morbidities known to accompany human twin gestations [12]. This is of potential importance, as twinning in human is an independent risk factor for the development of common perinatal morbidities and mortalities as prematurity, growth discordance, and twin-twin transfusion syndrome, all of which are recognized to render risk of adult obesity [13-15]. Despite these potential limitations, such early studies at relatively lower microbial fingerprinting resolution collectively suggest that that the structure of microbial communities is in some part influenced by their human host’s genome.

Human populations can be divided into mtDNA haplogroups based on SNPs scattered throughout the mitochondrial genome, reflecting mutations accumulated by the maternal lineage. Although there is association between ethnic groups and haplogroups, mtDNA variants are most broadly representative of ancient ancestral roots from tens of thousands of years ago. As people migrated to form isolated groups, small changes accumulated over generations to set genetic diversity. Ergo, haplogroups may be regarded as broadly representative of genomic ancestry but are neither the sole nor definitive ancestral tags and may exist in linkage with other nuclear DNA markers [16,17]. Given that race and ethnicity are delineated by haplogroup, and that mtDNA is more susceptible to DNA damage and acquires mutations across an individual’s lifetime at a higher rate than nuclear DNA, we hypothesized that mtDNA haplogroup and polymorphisms may be associated with variations in the human microbiome [2].

There are additional inherent characteristics of mtDNA, which make it an attractive candidate for host-microbiome association studies. Mitochondria derive from ancestral endosymbiont bacteria and have 16.5 kb circular double-stranded DNA molecules in multiple copies per cell (heteroplasmy). Consistent with their role as the generators of cellular ATP by oxidative phosphorylation, they play crucial roles in energy metabolism and apoptosis [18], and serve as the primary cellular source of reactive oxygen species [19]. The central role of mitochondrial proteins in cellular energy also makes mtDNA an ideal system for rapid human adaptation to new climate and dietary conditions [20]. The mtDNA genome encodes 13 protein-coding genes involved in respiration and oxidative phosphorylation, alongside two rRNAs and a complete set of 22 tRNAs that are important for protein synthesis (http://www.mitomap.org). Likely due to high levels of reactive oxygen species, lack of protective histones, and a limited DNA repair capacity, somatic mtDNA mutations (primarily in the D-Loop) are known to accumulate in individuals over time [21]. However, the majority of mtDNA variants are benign polymorphisms related to SNPs in the nuclear genome. The frequencies of these variants differ among populations and serve as the foundation of haplogrouping. The mutations in mtDNA have been related to a series of diseases related to neurological, muscular or metabolic disorder [22,23]. Recent population genetics studies also indicate the association of mtDNA variants with complex human diseases, such as Alzheimer’s, Parkinson disease and cancer [24,25]. Of interest to our studies, mitochondria have been recently suggested to play a pivotal role in the innate immune response [19,21,26-29].

In this study, we sought to provide initial evidence regarding the contribution of the host genome to its microbiome through robust interrogation of sequencing data and clinical metadata from the HMP [2-4]. Our aim was to determine whether there exists a significant association between haplogroups and mtDNA variants with microbiome taxonomic abundance and functional profiling.

## Results

### 16S and WGS profile construction

The Human Microbiome Project screened 554 individuals to enroll 300 subjects (149 males, 151 females, mean age 26, mean BMI 24, 20.0% racial minority and 10.7% Hispanic) [4]. A longitudinal sampling strategy yielded 11,174 primary specimens, from which 12,479 DNA samples were submitted to four centers for metagenomic sequencing. While the majority of the samples were targeted for 16S (V3-V5 region) profiling on 454 FLX Titanium platform [2,3], whole genome shotgun sequencing (WGS) data were generated for a subset of 681 samples. In this analysis, we utilized a robust cohort of samples from 89 individuals, which were sequenced at a single center and retained both 16S and WGS data. The Operational Taxonomic Unit (OTU) table [30] was generated using the HMP 16S pipeline with high stringency approach on V3 to V5 (v35) variable regions [2-4]. For each body site, OTU counts from samples belonging to individuals in the cohort were extracted to construct the OTU table (Additional file 1). The abundance of each taxonomic level (Phylum, Order, Family, Genus, Species) was binned and calculated based on the OTU table [2].

WGS was performed on the Illumina GAIIx platform. After identification and removal of human reads, microbial sequences were quality filtered and trimmed. The remaining sequences were aligned to protein families, and the abundance of each KEGG pathway and module [31] was generated to reveal the functional activities of each sample.

### Host SNP and haplogroup identification

Mitochondrial genomic sequences were extracted from whole genome sequencing of human samples by aligning with Cambridge Reference Sequence (NC_012920) through BWA (Burrows-Wheeler Aligner) [32]. High sequencing depth was achieved on all samples (mean 280X), thereby enabling a high degree of accuracy and precision of variant call. In total, 631 mitochondrial single nucleotide polymorphisms (mtSNP) from across the whole mitochondrial genome were identified by both GATK (Genome Analysis Tool Kit) [33] and SAMtools (Sequences Alignment/Map tool set) [34] as detailed in Methods.

Human mtDNA is characterized by variants, which in turn define haplogroups and polymorphisms. Mitochondria haplogroups are defined on the basis of haplogroup associating mtSNPs corresponding to the original RFLP (Restriction fragment length polymorphism)-defining loci [35]. All common European haplogroups (H, J, K, T, U, V, W, X, I), Asian haplogroups (B, F), African American haplogroups (L2, L3) and Mexican American haplogroups (A,C) were observed at high confidence with correlation to subject self-identified race and ethnicity [36]. Since the majority of enrolled subjects in HMP are Caucasian, the dominant haplogroups in our study are European haplogroups, especially HV and UK, and are in accordance with prior population-based cohorts [37].

### Association with haplogroup

**Figure 1:**
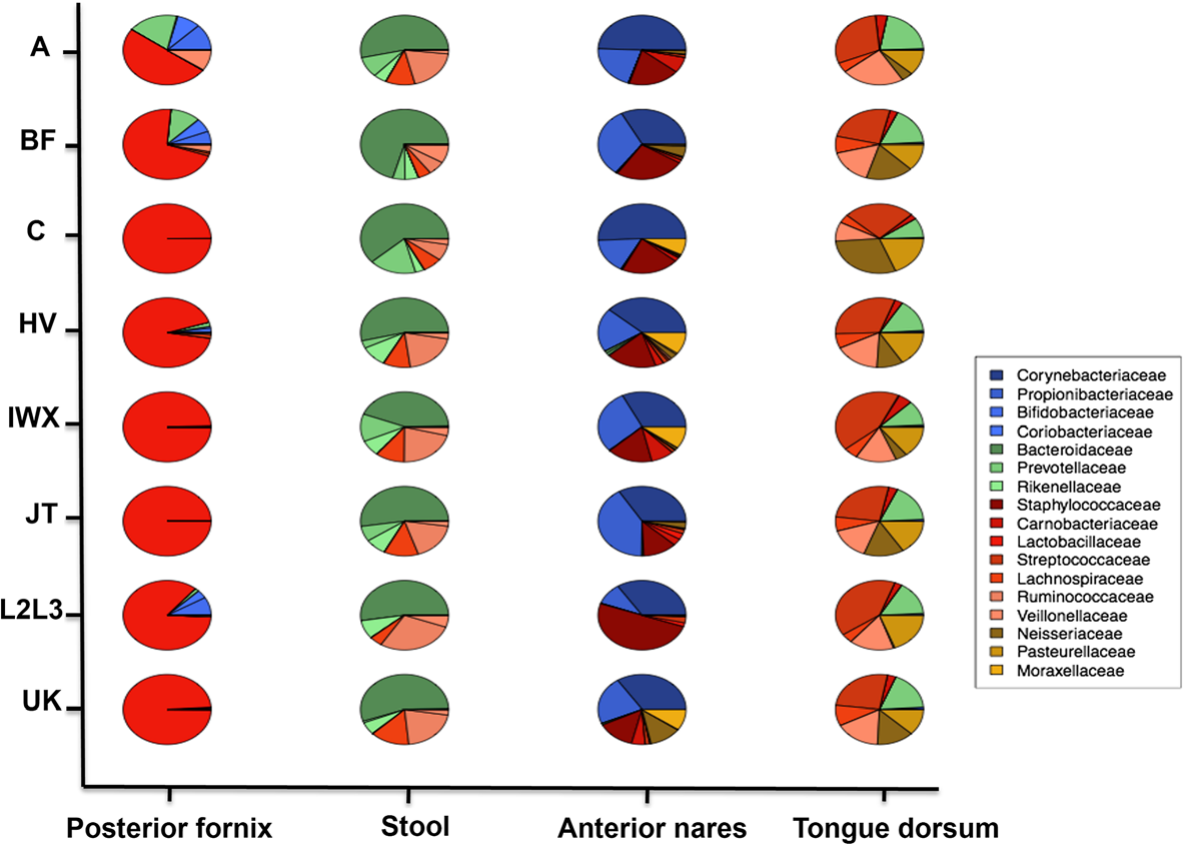
**Family level abundance distribution over haplogroups at various body sites.** The abundance of top five most abundant families at each body site (posterior fornix, stool, anterior nares, tongue dorsum) was selected to represent the community. Top families represent more than 90% of the total abundance for each body site.

In our first pass analysis, we tried to observe the difference of 16s-based microbiome community profiles among haplogroups at various body sites. OTUs from four body sites (stool, posterior fornix, anterior nares and tongue dorsum) representing four distinct body areas (gastrointestinal tract, vagina, skin and oral) were extracted separately to compare the distribution of top family level abundance in relation to haplogroup. To reduce the effect of low counts in less common haplogroups, based on the distance in the accepted haplogroup evolutionary tree [36], individuals were further binned into HV, JT, IWX, UK, A, BF, C and L2L3 groups [38]. As shown in Figure 1, although the overall family abundance at each body site is consistent with anticipated microbial community profiles [2], the abundance varies by virtue of host mtDNA haplogroup. *Lactobacillaceae* is decidedly the dominant taxa in the vaginal posterior fornix among most of subjects, yet the relative abundance of *Lactobacillaceae* is lower in haplogroup BF with high variance (mean 0.63, s.d. 0.42) compared with other haplogroups, such as haplogroup UK, which has a high and relatively stable *Lactobacillaceae* abundance (mean 0.96, s.d. 0.03) (Figure 1). Similarly, stool has a diverse taxonomic profile with familial taxa-projections dominated by the high abundance of *Bacteroidaceae*, *Ruminococcaceae* and *Lachnospiraceae*; however, a clear difference in *Bacteroidaceae* abundance between BF (mean 0.67, s.d. 0.15) and IWX (mean 0.29, s.d. 0.16) haplogroups is observed (Figure 1). The anterior nares has the highest number of families identified and the tongue dorsum samples are the most ecologically rich, the difference among haplogroups is also observable. There is marked difference of taxonomy abundance among individuals from various haplogroups, which may reflect the influence of maternal lineage on the microbiome.

**Figure 2:**
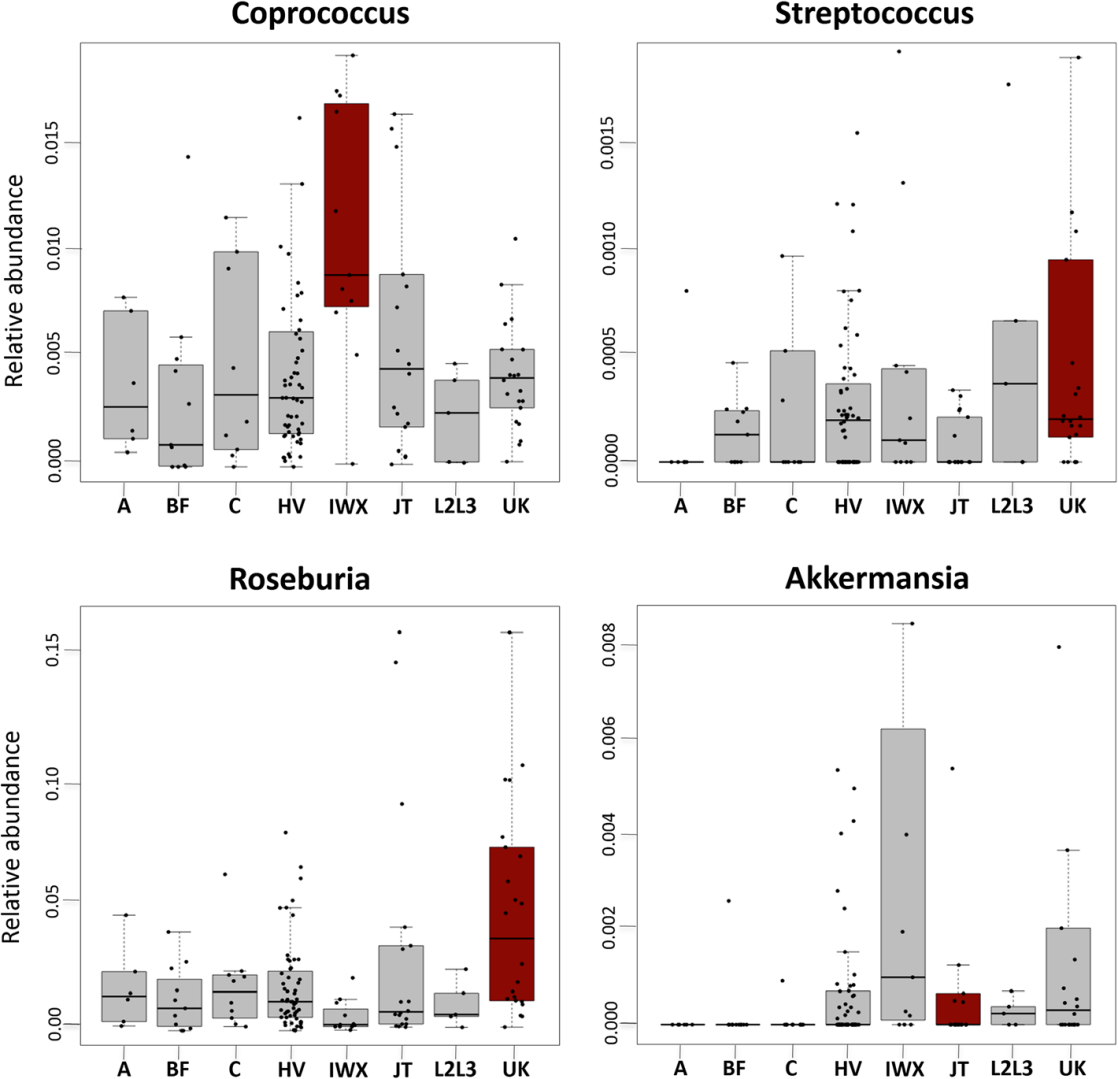
**The distribution of four genus level abundance over haplogroups in stool samples.** The Y-axis shows the relative abundance of the genus in corresponding haplogroups. The haplogroup box marked in red is the haplogroup associated with corresponding genus, which is discovered by multiple linear regression using genus level relative abundance as dependent variable and using haplogroup assignment, clinical metadata as independent variables. The number of samples in each haplogroup is parenthetically annotated, A(6), BF(11), C(10), HV(56), IWX(11), JT(18), L2L3(5), UK(22).

Given our initial observation described above, we next sought to interrogate genus-level association with haplogroups using stool samples. Recent research shows that niche selection, rather than neutral process, drives the assembly of the gut microbiome [39]. We therefore conducted multiple linear regression analyses to derive stool genus level abundance association with haplogroup together with other metadata. Assigning genus abundance as the dependent variable, we modeled host haplogroup and clinical metadata (including age, gender, race, BMI, blood pressure) as independent variables [2]. The linear regression modeling revealed a persistent association of genus abundance by virtue of haplogroups. For example, the IWX haplogroup has the highest abundance of *Coprococcus* (p value 0.016), while the BF haplogroup has the lowest abundance of *Coprococcus*. Similarly, the UK haplogroup demonstrated a preferential high abundance of *Roseburia* (p value 0.00025) and *Streptococcus* (p value 0.0086); JT haplogroup demonstrated a low abundance of *Akkermansia* (p value 0.0038) (Figure 2). Gender, BMI, age and blood pressure were neither modifiers of the haplogroup association, nor were they associated with projections of above genus abundance.

### Association with microbiome traits

Despite the evident complexity of the microbiome, two studies [40,41] have described microbial clusters dominated by *Bacteroides*, *Prevotella* and *Ruminococcus* in stool [40] and the relative dominance, absence, or presence of various species of *Lactobacillus* in vaginal samples [41]. However, the existence of discrete clusters is challenged by recent research [42], which demonstrates community gradients based on taxonomic abundance. As it was not a primary aim of our study to define or refute the existence of discrete clusters, we alternately tested the association of haplogroups with microbiome traits generated from gradients-based taxonomic abundance. We therefore investigated if individuals in our study of 89 subjects displayed identifiable traits based on taxonomic profile, then tested if these microbiome traits associated with the host haplogroup in the cohort.

**Figure 3:**
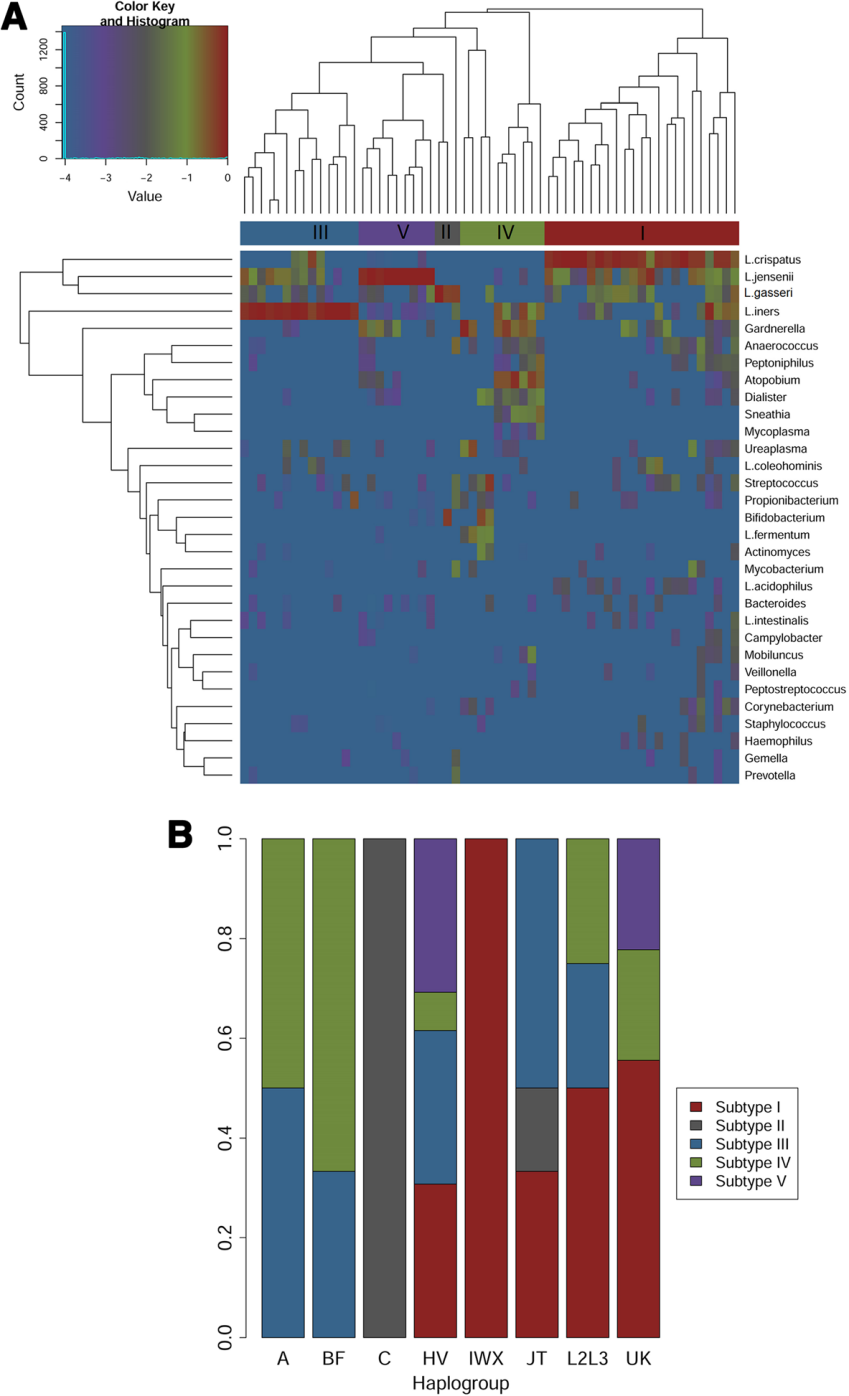
**Haplogroup associations with vaginal microbial community profiles. (A)** Heatmap of log10-transformed proportion of microbial taxa found in the posterior fornix samples (color key is indicated in the upper left corner). Subtype (trait) I-V assigned based on the species composition and abundance of vaginal microbial are shown on the top of the heatmap. **(B)** Vertical bar represent the distribution of each subtype I-V across haplogroups. The number of samples in each haplogroup is parenthetically annotated, A(2), BF(6), C(2), HV(26), IWX(4), JT(6), L2L3(4), UK(9).

Since the vaginal microbiome is dominated by *Lactobacillus* in most individuals, variance is primarily determined by the relative abundance of *Lactobacillus* spp [41]. Based on recent work on structure, function and diversity of the microbiome [2,3,43], posterior fornix taxonomy profiles were constructed using species abundance of *Lactobacillus* and genus abundance of other microbiota. All subjects could be assigned to one of groups primarily based on the abundance of specific *Lactobacillus* species, subtype I dominated by *L. crispatus*, subtype II dominated by *L. gasseri,* subtype III dominated by *L. iners*, subtype V dominated by *L. jensenii*, subtype IV not dominated by *Lactobacillus* (Figure 3A). These subtypes could then serve as traits for subsequent tests of association. As shown in Figure 3B, the distribution of subtype varies by virtue of host haplogroups (Chi-square test with Monte Carlo test of 2000 replicates, p value 0.001). 60% of individuals in Subtype IV, which interestingly is not dominated by *Lactobacillus* spp, have non-European haplogroups, while 90% of subtype I and all subtype V individuals are in European haplogroups (Figure 3B).

**Figure 4:**
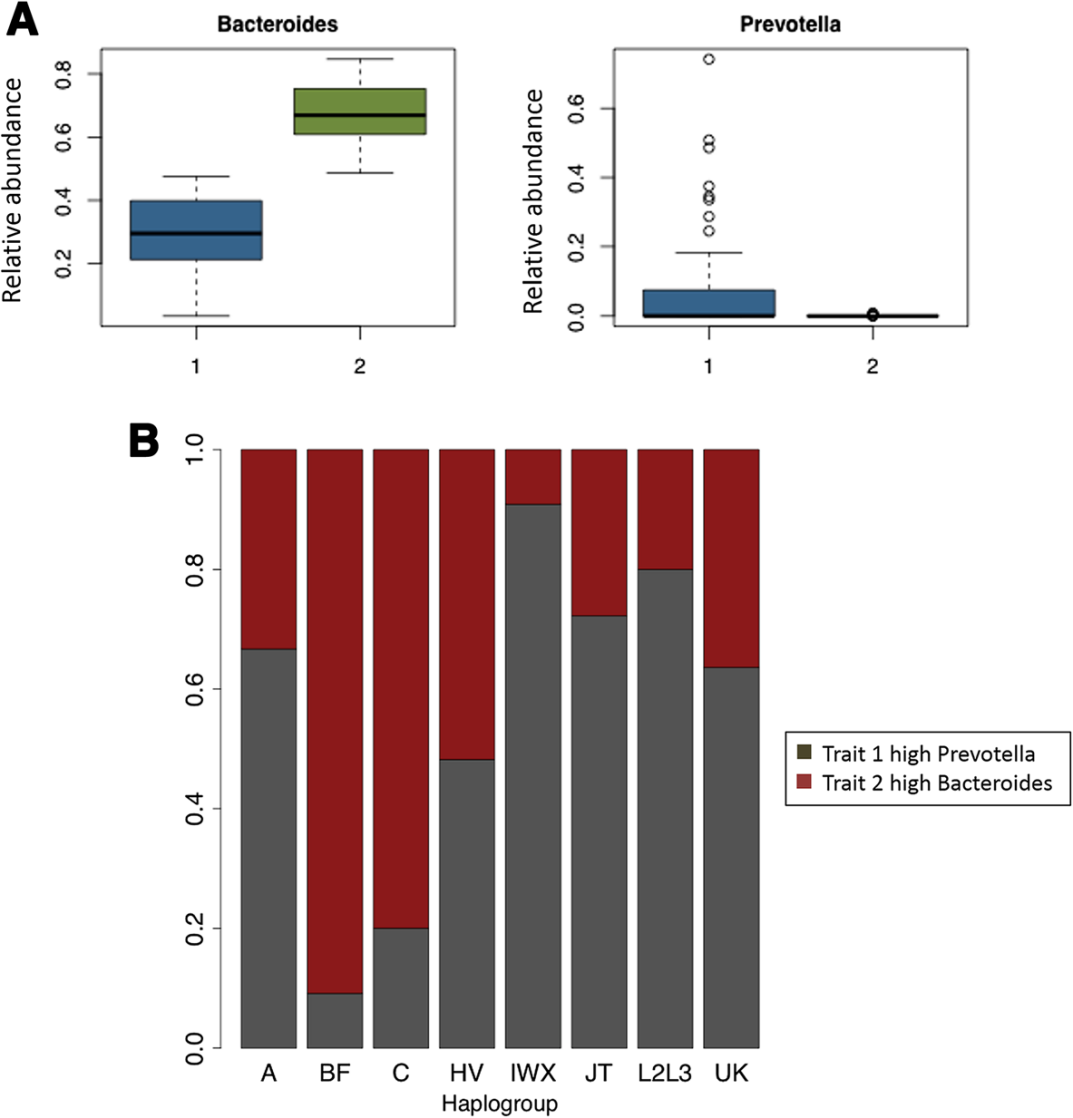
**Association of haplogroups with gut microbial community profiles. (A)** Relative abundance contributing to microbiome traits. The number on the X-axis indicates group 1 and 2. **(B) **Vertical bar represent the distribution of each group across haplogroup. The number of samples in each haplogroup is parenthetically annotated, A(6), BF(11), C(10), HV(56), IWX(11), JT(18), L2L3(5), UK(22).

Although PAM (Partitioning Around Medoids) clustering failed to identify enterotype-like clustering in our cohort, we observed that the variance of the relative abundance of *Bacteroides* and *Prevotella* are the highest among all genus level clades, with an observed trade-off between *Prevotella* and *Bacteroides* as previously described [6,42]. Samples were therefore assigned to one of two gut microbiome traits by virtue of their gradient *Bacteroides/Prevotella* ratio: group 1 with higher *Prevotella* abundance, and group 2 with higher *Bacteroides* abundance (Figure 4A). Akin to the vaginal microbiome trait projections, we again observed significant variation by virtue of host haplogroup (Chi-square test with Monte Carlo test of 2000 replicates, *p* value 0.002). The proportion of group 1 is significantly higher among the IWX haplogroup, and lower among the haplogroup BF (Figure 4B). The proportion of the two trait groups varies by large region-defined haplogroups, with bias by virtue of European haplogroups (HV, IWX, JT, UK) relative to Asian, Mexican American (BF, C) haplogroups. Of interest, we failed to observe a strong association of non-haplogroup defining mtSNPs to any microbiome trait.

### Association with SNP

To avoid potential confounding with subject-defined population stratification, we alternately stratified the cohort by haplogroup and performed SNP association exclusively on individuals classified into European haplogroups (namely H, J, K, T, U, V, W, X, I, 57 stool samples, 25 posterior fornix samples) [36]; this is as anticipated given that the majority of our subjects self-identified their ethnicity as Caucasian. Adjacent mtSNPs with similar profile could be represented by tagging SNPs instead of genotyping every individual mtSNP [44,45]. 158 tagging SNPs were identified using Tagger through Haploview [46] on mtSNPs identified in European haplogroups. These tagging mtSNPs are located in both regulatory and coding regions of the mitochondrial genome.

**Figure 5:**
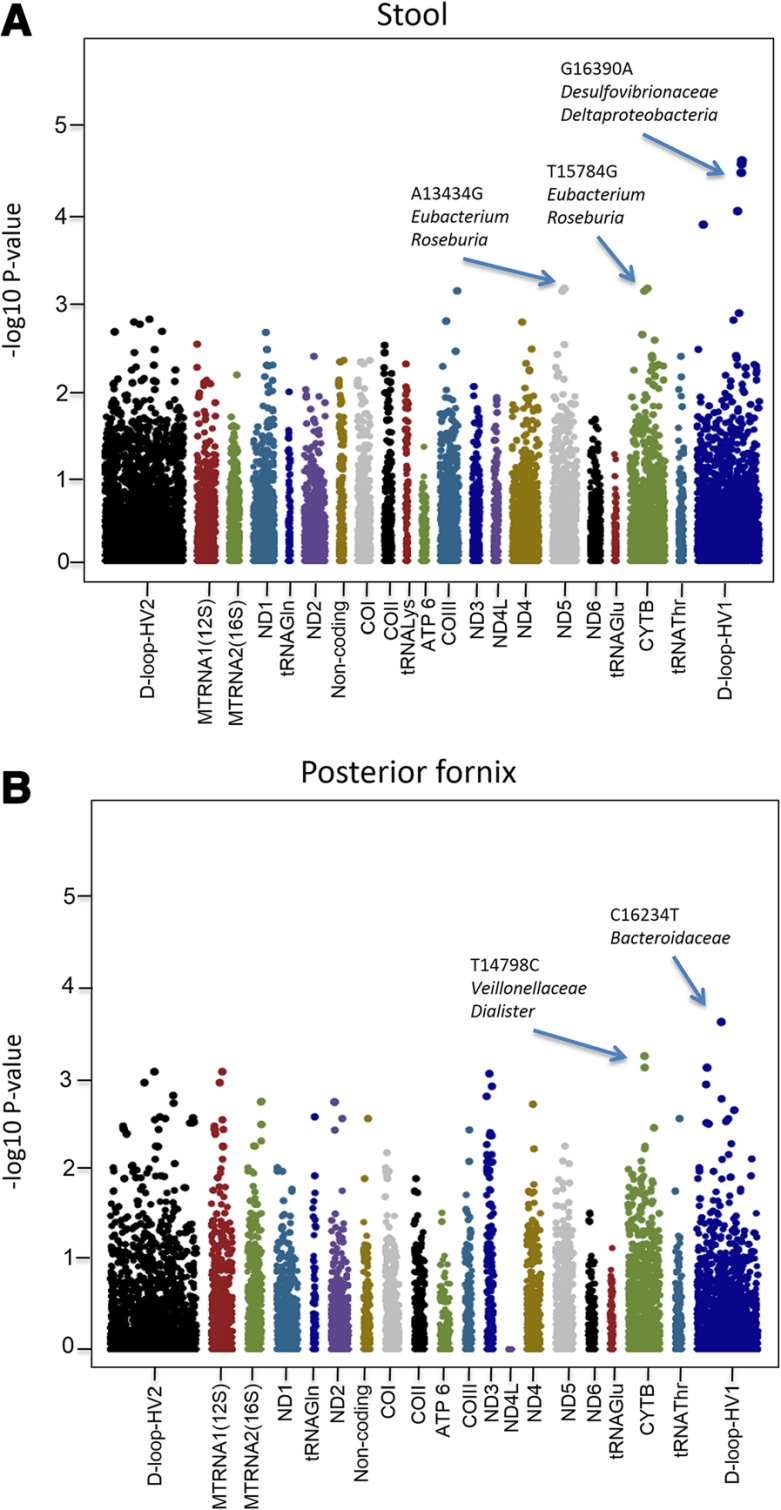
**Specific regions on mtDNA were shown to associate with stool (panel A) and posterior fornix (panel B) taxonomy profile.** -log10(pvalue) is used to represent the strength of association between specific mtSNP and clade. The –log10(pvalue) for each mtSNP on each clade were displayed in one Manhattan plot. The X-axis is labeled with mitochondrial gene names corresponding to their relative position on the chromosome. Significant association between mtSNPs and clade were marked in the plot.

The relative abundance of each clade was treated as a quantitative trait and tested for association with 158 tagging mtSNPs observed in the European cohort. In order to overcome the potential normality of continuous variable and rare allele testing, we adopted PLINK [47] quantitative trait associations with permutation to derive mtSNP associations. In gut samples, A13434G (q value 0.045) and T15784C (q value 0.045) are significantly associated with *Eubacterium* and *Roseburia*, which belong to *Clostridiale*. G16390A (q value 0.003) was observed in robust association with *Deltaproteobacteria* and *Desulfovibrionaceae*, which are both in the phylum of *Proteobacteria* (Table 1) (Figure 5A). In posterior fornix samples, a non-synonymous point mutation in cytochrome b, T14798C (q value 0.065), is associated with the *Veillonellaceae* family and one of its genus *Dialister* (Figure 5B). Overall, SNPs in the mtDNA genomic regions of ND5, CYTB and HV1 were in modest association with stool taxonomies. SNPs associated with microbiome profiles derived from the posterior fornix samples were distributed across the mtDNA genomic 12S region, ND3, CYTB, HV1 and HV2 regions.

**Table 1:** mtSNPs association with microbiome taxonomy profile in stool and posterior fornix samples

**Sample origin**	***SNP***	***Allele frequency***	***Rare SNP?***	***Location***	***Codon***	***p-value***	***q-value***	***Taxa associated***
**Stool 16S**								
	C16266T	1.82	N	HV1	N/A	0.0001	0.008	*Eggerthella*
	A13434G	0.41	Y	ND5	Met- > Met	0.0006	0.045	*Eubacterium,Roseburia*
	T15784C	3.143	N	CYTB	Pro- > Pro	0.0006	0.045	*Eubacterium,Roseburia*
	T16519C	59.72	N	D-loop	N/A	0.00007	0.011	*Alphaproteobacteria*
	G16390A	3.32	N	D-loop	N/A	0.00002	0.003	*Deltaproteobacteria, Desulfovibrionaceae*
**Posterior fornix 16S**								
	C16234T	3.59	N	HV1	N/A	0.0006	0.065	*Bacteroidaceae*
	T14798C	8.06	N	CYTB	Phe- > Leu	0.0006	0.065	*Veillonellaceae, Dialister*
	T1189C	3.85	N	12S	N/A	0.0005	0.024	*Veillonellaceae*

The results from whole genome sequencing of metagenomic samples were represented by KEGG pathway and module relative abundance for carriage patterns. Interestingly, we again observed a strong association between host mtSNP and gut carriage patterns (Table 2). Notably, the C3333T (q value 0.026) found in the ND1 coding region (synonymous mutation) exhibited a strong association with microbial metabolic pathways, including Phenylalanine metabolism and Styrene degradation (Table 2). In posterior fornix samples, a second synonymous point mutation T6776C (q value 0.049) in COI was observed in strong association with fatty acid metabolism.

**Table 2:** mtSNPs association with KEGG pathways and modules in stool and posterior fornix samples

**Sample origin**	***SNP***	***Allele frequency***	***Rare SNP?***	***Location***	***Codon***	***p-value***	***q-value***	***Pathway/module associated***
**Stool WGS**								
	C3333T	0.073	Y	ND1	Leu- > Leu	0.0001	0.026	*ko00360: Phenylalanine_metabolism*
	G11914A	7.87	N	ND4	Thr- > Thr	0.0001	0.014	*M00335: Sec(secretion) System*
**Posterior fornix WGS**								
	T6776C	2.55	N	COI	His- > His	0.0005	0.049	*ko00071: Fatty acid metabolism*
	A15924G	3.92	N	tRNAThr	N/A	0.0001	0.003	*M00193: Putative spermidine/ putrescine transport*

## Discussion

By enabling concomitant analysis of host genomic variants with their respective microbiome community profiles, we can begin to address a number of crucial gaps in our understanding. In this study, we observed significant associations among both mtDNA haplogroups and mtSNPs with microbiota taxa alongside their carriage patterns. These studies serve as robust initial population-based interrogations into host genome-microbiome associations, and further clarify previously described microbiome-ethnicity associations at the molecular level.

First, mtSNPs were identified for individuals in the HMP cohort with high confidence. Haplogroups were identified for each individual based on mtSNPs. Given the fundamental role of the mitochondrial genome in cellular metabolism, evidence has already accumulated that different human mtDNA lineages are functionally different [48,49]. mtDNA haplogroups are also thought to modify mitochondrial function because there is evidence that they are formed by adaptation to thermal environments [50] and subsequent natural selection [20]. It is clear that mitochondria haplogroup is more than a genomic marker. It reflects the ancestral difference in the human genome among the population. In publications from HMP consortium, association of clades and metabolic pathways with host properties, such as age, gender, BMI and race, have been preliminarily examined and reported [2,3]. A wide variety of taxa and metabolic pathways were differentially distributed by virtue of subject ethnicity at limited body sites. Although age was shown to be associated with several metabolic pathways and one skin clade, most other metadata (BMI, gender, etc) are generally modest and non-significant [2]. Ergo, other factors, such as host genetics, may play roles in shaping the host microbiome profile [2]. We acknowledge as a potential limitation to our study that while we observed an association between certain mtDNA SNPs and haplogroups with community microbial profiles, these associations may be more broadly representative of ancestral differences, which are in linkage with the mitochondrial genome.

Of interest to our data, Ravel *et. al.*[41] previously reported on the relationship of racial or ethnic background to vaginal bacterial community composition. Using a cohort of self-sampled subjects, they have previously reported on the proportion of vaginal subtypes, which significantly varied among Caucasian, Asian, African American and Latino subjects. Similarly, other investigators with the MetaHIT have shown that stool enterotype 1 is observed in strong association with self-identifying Japanese subjects [40]. Given that mtDNA haplogroups are the maternally derived genomic markers of race/ethnicity, our results have confirmed and extended these observations of others to molecular level.

Nevertheless, having defined mtDNA haplogroups existing in significant association with specific taxa, mtSNPs across mitochondria genome were further tested to identify regions with higher association. We observed several findings of potential relevance.

T14798C is a non-synonymous SNP that encodes an amino acid substitution of phenylalanine (Phe) to leucine (Leu). This mtSNP maps to cytochrome b and was observed in strong association with differential abundance of *Dialister* in the *Veillonellaceae* family specifically in the vaginal posterior fornix. This is of potential biological importance as the posterior fornix of the vagina is most proximal to the cervix and uterus and *Dialister* has been found in both amniotic fluid and placental tissue of women with preterm premature rupture of the amniotic sac [51]. There is long-standing racial and ethnic disparity in the risk of preterm birth [52].

Turning to the gut microbiome, A13434G is a synonymous mtSNP on ND5 and T15784C is a synonymous mutation also on cytochrome b. Although synonymous mutations are usually referred to as “silent”, increasing evidence demonstrates significant effects on transcription, splicing, and mRNA transport or translation, all of which would alter phenotype [53]. Both of the above synonymous mutations were observed in strong association with *Eubacterium* (belonging to *Clostridium* cluster IV) and *Roseburia* (*Clostridium* cluster XIVa). *Clostridial* clusters IV and XIVa are highly oxygen-sensitive anaerobes and produce butyrate along the GI tract, which is excreted in feces [54]. Notably, decreased abundance in members of the butyrate-producing *Clostridial* clusters IV and XIVa have been reported in the gut of inflammatory bowel disease (IBD) patients, another prevalent human disease which displays variable risk by virtue of race and ethnicity [55]. Functionally extending these observations to the microbiota metabolic profile, several mtSNPs mapping to the mitochondrial encoded NADH dehydrogenase genes were observed in significant association with pathways in amino acid metabolism and protein secretion system from stool samples. It has long been debated whether amino acid derived molecules produced by intestinal bacteria affect host health by regulating host immunity and cell function, or alternately by varying microbial composition and community metabolism [56]. Our findings suggest that the host mtDNA variants in key redox pathways inherently define the gut microbiome, which in turn will structure their community and carriage patterns. This is consistent with long standing demonstrations that in microbiota, protein secretion transport effector molecules function from the interior to exterior. Moreover, protein secretion is known to play a central role in modulating the interactions of bacteria with their environments, particularly when symbiotic bacteria interact with host cellular constituents [57].

The primary role of the mitochondria is to produce energy for the cell. Overall, we observed more clades and metabolic pathways to occur in significant association with mtSNPs mapping to genomic loci encoding for electron transport. The mitochondrial respiratory chain, which is comprised of five multi-subunit protein complexes, carries out oxidative phosphorylation. During oxidative phosphorylation, mitochondrial enzyme complexes generate an electrical charge on either side of the inner mitochondrial membrane through transferring of electrons (membrane potential). This potential difference in electrical charge provides the energy for ATP production. NADH dehydrogenase genes encoded by mtDNA comprise complex I, which is responsible for the first step in the electron transport process. Mitochondrial cytochrome b is a subunit of complex III, which passes electrons to cytochrome c, which relays them to complex IV; three of protein subunits of complex IV are encoded by mitochondrial originated cytochrome c oxidase.

Our observed mutations on mitochondrial genes are known to disrupt normal activity of the electron transport chain, which will affect the production of ATP and increase the production of reactive oxygen species (ROS)[58]. ROS have important roles in cell signaling and homeostasis, but excessive amount of ROS could also cause significant damage to cell structures [59]. In human disease studies, oxidative stress is involved in the development of many diseases, including cancer [60], Parkinson’s disease, Alzheimer’s disease [61], and inflammatory bowel disease [62]. In addition to generating cellular energy, mitochondria are also involved in cell differentiation, cell death, as well as the control of cell cycle and growth [63]. It is also well established that the mitochondrial genomes are dynamic structures whose quantity and quality alter in response to cellular oxidative and metabolic demands. Although the mitochondrial genome is small, mtDNA encodes genes essential to perform the above functions and maintain symbiotic host-microbiome relationships.

In sum, the regulation of metabolic function is highly conserved in higher eukaryotes and is essential to the establishment of microbial communities. Although pathogenic mutations on mtDNA result in severe disease, milder mutations may have subtle phenotypic consequences. Ergo, the mtDNA variations among individuals in the HMP cohort may still render different microbiome profiles, possibly through inflammation response to different levels of ROS activity [58,64]. Although the detailed mechanism awaits further analysis, individuals who have mtSNPs associated with haplogroups discovered in our study may show differences in their capability of energy metabolism and thus affect their own microbiome profile. We speculate that larger population-based and prospective analyses, which would enroll both healthy and disease-afflicted subjects, will provide further robust evidence for our initial associations proposed herein.

## Conclusions

We have described our approach to leverage sequencing data from the HMP to demonstrate significant association between the human microbiome with host mtDNA variants (haplogroup and nucleotide polymorphisms). We observe that among stool and vaginal posterior fornix samples, several haplogroups and mtDNA variants show significant association with specific microbiota and their community structure traits. Delving deeply into both mtSNPs and metagenomic-derived carriage patterns, we have further shown that our observed associations between host and microbe are of likely functional relevance. Given the long-standing described role of mitochondria in cellular metabolism and oxidative stress, and emerging data describing its role in innate immunity, our findings may be of likely high significance. In sum, these data provide initial evidence for the host mitochondrial genome influencing the structure of its microbiome, and underscore the capacity for metagenomics to explore host-microbe interactions.

## Methods

### Subjects

Our initial study consists of 89 subjects with self identified NIH-defined race (74 Caucasians, 7 Asians, 4 African American and 4 Mexican American, 43 female, 46 male) and ethnicity (Hispanic versus non-Hispanic) in the Human Microbiome Project recruited at Houston, Texas and St. Louis, Missouri. Subjects were all healthy individuals and were sampled one to three times at 15 (male) or 18 (female) body habitats following a common sampling protocol [4]. The subjects were between 18-40 (mean 26 s.d. 5) years old to minimize the variability due to growth and aging. The mean of BMI is 24 (s.d. 4). For 16S, there are 139 stool samples from 81 individuals and 59 posterior fornix samples from 37 individuals in the cohort. For WGS, there are 116 stool samples from 79 individuals and 50 posterior fornix samples from 37 individuals available. The HMP study shows that within-subject variation over time is consistently lower than between-subject variation at both taxonomy and metabolic level [2]. Since each individual’s microbial community is stable over time relative to the population as a whole, the microbial clades association of clinical metadata analysis in HMP includes data from all visits. Only the data from first visit was used for mtSNPs association analysis in our study.

The 16S and WGS data used in this study were downloaded from hmpdacc.org. The detailed procedure for data processing is described in the HMP publication. Briefly, raw V3-V5 region 16S sequences were demultiplexed using QIIME [65]. OTU picking (including error correction, chimera checking through QIIME and clustering via UCLUST [66]) was performed by OTUPipe on V3-5 region. Taxonomy was assigned using the RDP classifier 2.2 [67]. For metabolic reconstruction, the HMP Unified Metabolic Analysis Network (HUMAnN) [68] was used to infer KEGG based community functions from WGS reads. The OTU counts, pathway/module abundance for subjects in our study were extracted from summary OTU table and abundance files (HMQCP for 16S, HMMRC for WGS metabolic reconstruction).

### Variants identification

Sequence alignment, quality control, and variant calling were performed with BWA (Burrows-Wheeler Aligner) [32], SAMtools [34], Picard and the Genomic Analysis Toolkit (GATK) [33]. In this study, we utilized the small percentage of human reads from whom the microbiome samples were collected. The whole genome sequencing data without removal of human sequences could be obtained from dbGaP (phs000228.v2.p1). Prior to variant calling, mitochondrial genomic sequences were extracted from whole genome sequencing of human microbiome samples by aligning with Cambridge Reference Sequence (NC_012920) through BWA. SAMtools were used to convert, sort, and index the aligned data files. Picard was then used to identify and remove duplicate reads from each lane. Variants were identified with GATK’s variant detection tools. Following base quality recalibration, indel realignment and unified genotyper, the single-nucleotide variants were filtered for the removal of low-quality variant calls with GATK’s VariantFiltrationWalker tool. In addition to GATK, SAMtools was also used to call targeted bases, and any base call that deviates from reference base was regarded as a potential variation.

### Haplogroup analysis

Based on published references [23], PhyloTree [69] and the MITOMAP [36] database, genotypes of several mtSNPs were combined to construct the haplogroups. The most common European mtDNA haplogroups include H, HV, I, J, K, T, U, V, W and X. 70 individuals were identified as European haplogroups. Typical haplogroups for Asian, African American, Mexican American and the rest of Caucasian were identified as A, B, C, F and L2. 19 individuals were classified into non-European haplogroups.

### Microbiome trait identification

Since the vaginal microbiome is dominated by one or more species of *Lactobacillus*, the representative sequences of each OTU in the vagina microbiome was aligned to Greengenes (4feb2011 version) [70] through blast to achieve species level assignment. Greengenes maintains a consistent multiple-sequence alignment of both archaeal and bacterial 16S sequences to facilitate this process. Major *Lactobacillus* species in the vaginal microbiota (*L.crispatus, L.iners, Ljensenii and L.gasseri*) were further checked for alignments. OTUs with assignment to *Lactobacillus* species and other genera based on RDP classifier were normalized (OTU relative abundance were combined based on taxonomy assignment. The abundance of *Lactobacillus* species and other genera abundance sum to 1.) and then clustered through complete linkage hierarchical clustering using R packages. Subjects were assigned to vaginal community memberships according to dendrogram in the heat map (Figure 3A).

The reconstructed OTU table of stool samples was normalized (The OTU table column sum to 1.) and clustered using partitioning around medoids (PAM) clustering over Euclidian distance. The optimal number of clusters is two, which is validated by silhouette validation technique (silhouette score > 0.5). Group 1 has higher abundance of *Prevotella*, group 2 has higher abundance of *Bacteroides*. Our assignment of groups is generally consistent with Wu *et al*[71].

### Haplogroup associated mtSNPs

Since groups of mtSNPs were used for identifying haplogroups, it remained a formal possibility that some other non-haplogroup identifying mtSNPs may be also linked to certain specific haplogroup. These haplogroup-associated mtSNPs may be not as informative as non-haplogroup associated SNPs. To identify haplogroup associated SNPs among European, information from two sources was used. Saxena et al. [37] cataloged all common sites in mtDNA (excluding the control region) from 928 Europeans. 144 sites has a frequency of >1% in these individuals. 64 tagging SNPs were identified as being necessary to tag common mitochondrial variation as well as nine haplogroups (H, V, J, T, U, K, I, W, X) with an r^2^ (squared correlation of the alleles at two SNPs) of 0.8 using Tagger. Based on Table four in Soxena et al., 69 SNPs out of 144 SNPs (19 tagging SNPs out of 64 tagging SNPs) are identified as potential haplogroup associated SNPs. The other source used is phylotree.org, which includes updated comprehensive phylogeny of global human mtDNA variation and haplogroups. SNPs used for assigning haplogroups down to one level lower than the nine haplogroups mentioned above were identified as potential haplogroup associated SNPs. Only SNPs identified from both sources are marked as haplogroup associated SNPs.

### Association analysis

Metadata for HMP individuals are available from dbGaP. All OTU counts in the cohort stool or posterior fornix communities were normalized to relative abundance, where the OTU table columns sum to 1, and relative abundance for each clade was inferred based on the RDP annotation (The abundance of each genus in the same sample sum to 1). There are 109 genera identified from HMP stool samples and 51 genera identified from HMP posterior fornix samples. To avoid bias caused by rare genus, genus appears in less 5% of samples were not included in association analysis. Haplogroup association with quantitative trait was done through multiple linear regression, with clade abundance as dependent variable, and each identified haplogroup (with other haplogroups as one group), BMI, gender, race, blood pressure and age as independent variables. The linear regression was conducted in R. The significance of each independent variable was adjusted for multiple comparisons using R package qvalue [72] with a list of p-values resulting from testing on each clade as input and estimated q-value as output. The q-value measures the false discovery rate of the test.

Since several SNPs were correlated with other SNPs, we used Tagger implemented through haploview [46] to find tagging SNPs which have pairwise values r^2^ > =0.8. Those mtSNPs tagged by other mtSNPs were not included in the association analysis. mtSNP association analysis with each quantitative trait (either arcsin square root transformed clad abundance or pathway/module abundance) is carried through PLINK [47]. Quantitative trait association with adaptive permutation procedure using default parameters. Given the property of our data, permutation test could relax the assumption about normality of continuous phenotypes and dealing well with rare alleles, as well as small sample size. To control for multiple testing, empirical p-value for each SNP from PLINK was adjusted for multiple comparisons through R package qvalue. SNPs with a q-value less than 0.05 are considered significant.

### Data availability

The 16S and WGS data used in this study were downloaded from hmpdacc.org (HMQCP for 16S, HMMRC for WGS metabolic reconstruction). Since there are large amount of human sequences in the whole genome sequencing data, the sequencing data without removal of human sequences could be only obtained from dbGaP (phs000228.v2.p1). Metadata for HMP individuals are also available from dbGaP.

## Abbreviations

BMI: Body mass index; CMM: Core measurable microbiota; FDR: False discovery rate; GATK: Genomic analysis toolkit; HMP: Human microbiome project; mtDNA: mitochondrial DNA; mtSNP: mitochondrial single nucleotide polymorphisms; OTU: Operational taxonomic unit; PAM: Partitioning around medoids; RFLP: Restriction fragment length polymorphism; WGS: Whole-genome shotgun.

## Competing interests

The authors declare no competing financial interests.

## Authors’ contributions

KA and JV are investigators with the Human Microbiome Project consortium. KA, JM, JV are currently funded through NICHD and/or the Burroughs Welcome Fund Preterm Birth Initiative on microbiome profiling in maternal health, pregnancy, and parturition. KA is a Maternal-Fetal Medicine specialist, and PB is an expert in mitochondrial DNA variation and mtDNA genomic sequencing and assembly with a focus on mitochondrial disorders and metabolism. JM, CC, XQ and AM are bionformaticians with the Bioinformatics Research Lab and the Human Genome Sequencing Center at Baylor College of Medicine. All authors read and approved the final manuscript.

## Supplementary Material

Additional file 1 **OTU tables and KEGG module/pathway tables.** This spreadsheet has 8 tabs. The OTU tables of posterior fornix, stool, anterior nares, tongue dorsum and HUMAnN generated KEGG module/pathway activity files for posterior fornix, stool samples used in the study were provided. Click here for file
